# Microwave ablation combined with EGFR-TKIs versus only EGFR-TKIs in advanced NSCLC patients with EGFR-sensitive mutations

**DOI:** 10.18632/oncotarget.18083

**Published:** 2017-05-23

**Authors:** Zhigang Wei, Xin Ye, Xia Yang, Aimin Zheng, Guanghui Huang, Wenhong Li, Jiao Wang, Xiaoying Han, Min Meng, Yang Ni

**Affiliations:** ^1^ Department of Oncology, Shandong Provincial Hospital Affiliated to Shandong University, Jinan 250021, Shandong Province, China

**Keywords:** epidermal growth factor receptor, tyrosine kinase inhibitors, non-small cell lung cancer, microwave ablation, progression free survival

## Abstract

We conducted this retrospective study to investigate whether microwave ablation (MWA) of primary tumor sites plus epidermal growth factor receptor-tyrosine kinase inhibitors (EGFR-TKIs) could improve survival in advanced non small cell lung cancer (NSCLC) with EGFR mutations. MWA was conducted at the primary tumor sites, followed by EGFR-TKIs in the MWA plus EGFR-TKIs group. EGFR-TKIs were administered until disease progression or intolerable toxicity. The primary endpoint was progression-free survival (PFS); secondary endpoints were overall survival (OS) and objective response rate (ORR). A total of 58 patients were recruited, including 34 in the MWA plus EGFR-TKIs group and 24 in the EGFR-TKIs group. No significant difference in ORR was observed with MWA treatment (61.8% vs. 45.8%, p = 0.230). Patients treated with MWA plus EGFR-TKIs failed to show superior survival in either PFS (13.2 months vs. 11.6 months, p = 0.640) or OS (39.8 months vs. 20.4 months, p = 0.288). MWA was not an independent prognostic factor for PFS or OS. MWA of primary tumor sites plus EGFR-TKIs demonstrated no survival advantage compared with EGFR-TKIs alone in advanced NSCLC patients with EGFR sensitive mutations. MWA should not be recommended for unselected patients with EGFR-sensitive mutations.

## INTRODUCTION

EGFR sensitive mutations, especially in-frame deletions in exon 19 and a point mutation in exon 21 (L858R), are present in 30–40% of non-small cell lung cancer (NSCLC) patients and respond well to EGFR-TKIs such as gefitinib and erlotinib. It is estimated that the ORR ranged from 60% to 83%, with a median PFS of 8 to 11 months. [1–5]

Patients who respond to EGFR-TKIs will develop resistance eventually. Several mechanisms had been clarified; among them, an EGFR T790M mutation in exon 20 [6–9] and c-MET [10, 11] amplifications have been explored widely, and account for 50% and 25% of all resistant mechanisms, respectively. What is more, PIK3CA mutation, [12] ERBB2 amplification, [13] HGF overexpression, [14] AXL activation, [15] epithelial-mesenchymal transition, and pathology type transformation, especially adenocarcinoma transformation into small cell lung cancer, have also been reported as causes of secondary resistance to EGFR-TKIs. [16–18]

According to the progression of EGFR-TKIs, secondary resistance can be clarified into three types: intracranial disease progression, development of asymptomatic oligometastases, and symptomatic disease progression. [19, 20] For the former two types, EGFR-TKIs could be continued after local therapy is administered. [21, 22]

For patients with intracranial progression, both whole-brain radiation therapy (WBRT) and stereotactic radiotherapy (SRT) could be treatment regimens. [20–22] For patients with oligometastases other than intracranial metastases, radiation therapy or thermal ablation could be applied. [20, 23–25] Ni et al. showed that patients with extra-central nervous system oligoprogressive disease had a median PFS of 8.8 months after microwave ablation (MWA), which was significantly different when compared with a transformation to platinum-based doublet chemotherapy. [25]

In previous studies, we verified that advanced NSCLC could benefit from a combination of MWA at primary tumor sites and platinum-based doublet chemotherapy. [26] What is more, a significant difference in PFS was also observed when compared with chemotherapy alone. [27] A recent phase II prospective, randomized, controlled clinical trial verified that local consolidative therapy with maintenance therapy for patients with three or fewer metastases from NSCLC (those who benefit from first-line systematic therapies) and demonstrated improved PFS when compared with maintenance therapy alone. The median PFS were 11.9 months and 3.9 months for consolidative plus maintenance therapy and maintenance therapy alone, respectively. [28] Local therapy including radiation and MWA in combination with systematic therapies improve survival in advanced NSCLC. Therefore, we conducted this retrospective study to determine whether advanced NSCLC patients with EGFR-sensitive mutations could benefit from MWA at primary tumor sites plus EGFR-TKIs when compared with EGFR-TKIs alone.

## RESULTS

### Patient characteristics

From January 25, 2010 to May 19, 2016, 58 patients were enrolled, of whom 34 were in the MWA plus EGFR-TKIs group and 24 were in the EGFR-TKIs group. In the MWA plus EGFR-TKIs group, 26 were women, 13 were aged 65 years or older, 33 had adenocarcinoma histology and an ECOG PS of 1, and 28 were nonsmokers. EGFR mutations included 18 19Del mutations and 19 exon 21 L858R mutations (including 3 patients with both 19Del mutations and L858R mutations). Nineteen patients were treated with EGFR-TKIs as a first-line therapy and 15 patients were treated with EGFR-TKIs as a post-first-line therapy. The primary tumor size ranged from 0.8 to 9.0 cm, with a mean of 3.7 cm. Most patients (23 patients, 67.6%) underwent ablation with two antennas and the typical ablation energy was 70 W (23 patients, 67.6%). In the EGFR-TKIs group, 13 were women aged 65 years or older, 23 had adenocarcinoma histology, 21 had an ECOG PS of 1, and 17 were nonsmokers. EGFR mutations included 16 19Del mutations and 10 exon 21 L858R mutations (including two patients with both mutations). Sixteen patients were treated with EGFR-TKIs as a first-line therapy, and 8 patients were treated with EGFR-TKIs as a post-first-line therapy. The primary tumor size ranged from 1.6 to 8.8 cm, with a mean of 3.9 cm. Baseline characteristics of the enrolled patients are shown in detail in Table 1.

**Table 1:** Baseline characteristics of 58 enrolled patients

		MWA+EGFR-TKIs		EGFR-TKIs	
		Number	Percent(%)	Number	Percent(%)
Gender					
	Male	8	23.5	11	45.8
	Female	26	76.5	13	54.2
Age	58.9(29-85)		62.0(38-79)		
	≥65	13	38.2	11	45.8
	<65	21	61.8	13	54.2
Smoking history					
	Non-smokers	28	82.4	17	70.8
	Smokers	6	17.6	7	29.2
ECOG					
	0	1	2.9	3	12.5
	1	33	97.1	21	87.5
Pathology					
	Adenocarcinoma	33	97.1	23	95.8
	Non-adenocarcinoma	1	2.9	1	4.2
Stage					
	IIIB	3	8.8	1	4.2
	IV	31	91.2	23	95.8
EGFR sensitive mutations					
	19Del	18*	39.4	16^#^	66.7
	L858R	16	60.6	8	33.3
Primary tumor size					
	Mean	3.7		3.9	
	Range	0.8-9.0		1.6-8.8	
Primary tumor size					
	≥3.5cm	15	44.1	12	50.0
	<3.5cm	19	55.9	12	50.0
Primary tumor site					
	Right lung	20	58.8	15	62.5
	Left lung	14	41.2	9	37.5
Primary tumor site					
	Upper and middle lobe	12	35.3	21	87.5
	Lower lobe	22	64.7	3	12.5
Power of MWA					
	60W	11	32.4		
	70w	23	67.6		
Time of MWA					
	Mean	11.5 minutes			
	Range	3-28.5 minutes			
EGFR-TKIs regimen					
	1^st^-line	19	55.9	16	66.7
	2^nd^ and post-2^nd^ line	15	44.1	8	33.3
EGFR-TKIs type					
	Gefitinib	32	94.1	18	75.0
	Erlotinib	2	5.9	6	25.0
Response					
	CR+PR	21	61.8	11	45.8
	SD+PD	13	38.2	13	54.2
Treatment post-TKIs					
	No	20	58.8	16	66.7
	Yes	14	41.2	8	33.3
Treatment post-TKIs					
	Chemotherapy	4	28.6	3	42.9
	Previous TKIs	8	57.1	4	57.1
	Other TKIs	2	14.3	0	0.0

*Three patients had both 19Del and L858R mutations.

^#^Two patients had both 19Del and L858R mutations.

### Response to MWA and EGFR-TKIs

Complete ablation was achieved in 29 (85.3%) of the 34 total patients in the MWA plus EGFR-TKIs group. The ORRs to EGFR-TKIs in the MWA plus EGFR-TKIs and EGFR-TKIs groups were 61.8% (21/34) and 45.8% (11/24) (p = 0.230), respectively.

### The correlation between MWA and survival

Until November 19, 2016, with a median follow-up of 19.3 months (range, 6 to 52 months), 35 patients progressed, including 21 in the MWA plus EGFR-TKIs group and 14 in the EGFR-TKIs group. Sixteen patients died, of whom 9 and 7 were in the MWA plus EGFR-TKIs group and EGFR-TKIs group, respectively.

**Figure 1:**
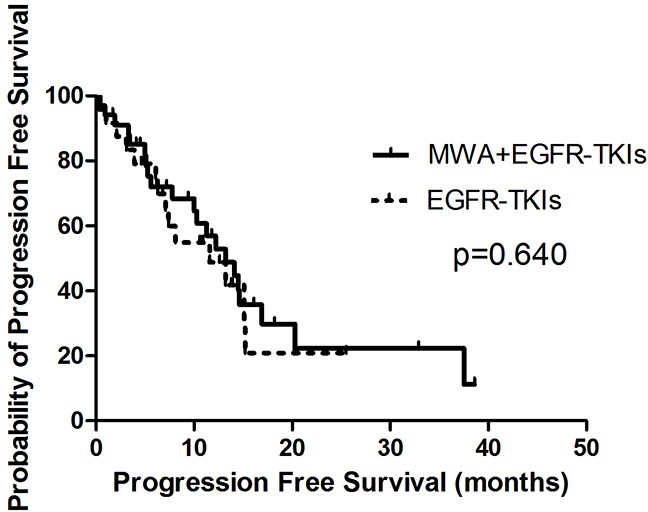
Kaplan-Meier estimates of PFS in 58 patients The median PFS of patients treated with MWA plus EGFR-TKIs was 13.2 months (95%CI, 9.0-17.5 months), and those received EGFR-TKIs was 11.6 months (95%CI, 4.7-18.5 months).

Patients treated with MWA plus EGFR-TKIs failed to show a survival advantage when compared with EGFR-TKIs only, with PFS durations of 13.2 months (95% confidence interval [CI], 9.0–17.5 months) and 11.6 months (95% CI, 4.7–18.5 months, p = 0.640), respectively (Figure 1). Patients with adenocarcinoma had longer PFS in comparison with non-adenocarcinoma NSCLC (13.2 months, 95% CI, 9.8–16.6 months vs. 0.4 months, 95% CI, 0.2–0.5 months, p = 0.000). The response to EGFR-TKIs had a tendency to predict PFS (CR + PR vs. PD + SD, 14.6 months [95% CI, 12.4–16.9 months] vs. 10.0 months [95% CI, 3.8–16.3 months], p = 0.067) (Table 2).

**Table 2:** Univariant analyses of progression free survival and overall survival

		PFS(ms)	95%CI	p	OS(ms)	95%CI	p
Gender				0.053			0.140
	Male	7.4	4.2-10.7		21.4	12.4-30.4	
	Female	14.1	12.1-16.1		39.8	13.8-65.7	
Age				0.631			0.490
	≥65	14.1	9.8-18.4		23.5	18.0-29.0	
	<65	12.2	7.9-16.4		32.5	24.4-40.6	
Smoking history				0.403			0.540
	Non-smokers	13.2	10.2-16.1		31.8	24.6-38.9	
	Smokers	10.3	5.3-23.3		23.1	25.1-38.0	
ECOG				0.373			0.720
	0	20.1	7.4-32.7		28.2	19.9-36.5	
	1	14.8	10.7-18.8		31.1	29.9-42.6	
Pathology				0.000			0.000
	Adenocarcinoma	13.2	9.8-16.6		16.2	12.2-20.3	
	Non-adenocarcinoma	0.4	0.2-0.5		0.4	0.2-0.5	
Stage				0.692			0.938
	IIIB	10.0	4.1-16.0		25.6	13.6-37.6	
	IV	13.1	10.2-16.1		31.2	24.5-37.9	
EGFR mutation				0.599			0.385
	19Del	11.6	6.2-17.0		36.3	29.9-41.6	
	L858R	13.2	10.6-15.7		27.7	19.9-35.5	
MWA				0.640			0.288
	No	11.6	4.7-18.5		20.4	11.3-29.5	
	Yes	13.2	9.0-17.5		39.8	8.3-71.2	
EGFR-TKI regimen				0.501			0.975
	1^st^-line	14.1	10.1-18.2		30.7	23.0-38.4	
	2^nd^ and post-2^nd^ line	12.2	3.6-20.8		31.1	22.0-40.3	
EGFR-TKI type				0.981			0.936
	Gefitinib	13.2	10.3-16.1		30.3	23.4-37.2	
	Erlotinib	7.1	0.0-26.8		34.3	21.4-47.2	
Response				0.067			0.042
	CR+PR	14.6	12.4-16.9		36.7	28.2-45.3	
	SD+PD	10.0	3.8-16.3		25.2	17.5-33.0	
Treatment post-TKIs				0.147			
	No	5.0	3.1-7.0		11.4	7.7-15.1	
	Yes	7.8	2.9-12.7		39.7	5.5-74.0	

**Figure 2:**
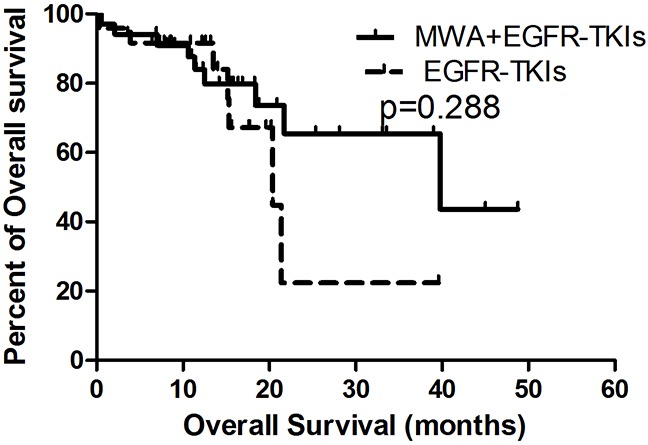
Kaplan-Meier estimates of OS in 58 patients The median OS of patients treated with MWA plus EGFR-TKIs was 39.8 months (95%CI, 6.3-71.2 months), and those received EGFR-TKIs was 20.4 months (95%CI, 11.3-29.5 months).

OS was similar in both groups (MWA plus EGFR-TKIs group vs. EGFR-TKIs group, 39.8 months [95% CI, 6.3–71.2 months] vs. 20.4 months [95% CI, 11.3–29.5 months], p = 0.288; Figure 2). Patients with adenocarcinoma had better OS in comparison with non-adenocarcinoma NSCLC (16.2 months [95% CI, 12.2–20.3 months] vs. 0.4 months [95% CI, 0.2–0.5 months], p = 0.000). Patients who achieved an ORR (CR + PR vs. PD + SD, 36.7 months [95% CI, 28.2–45.3 months] vs. 25.2 months [95% CI, 17.5–33.0 months], p = 0.042) and received treatment after progression with EGFR-TKIs (treatment post-EGFR-TKIs vs. no treatment post-EGFR-TKIs, 39.7 months [95% CI, 5.5–74.0 months] vs. 11.4 months [95% CI, 7.7–15.1 months], p = 0.000) also showed a survival advantage (Table 2).

In the multivariate analyses of PFS and OS, MWA was not a significant prognostic factor, and the corresponding p-values were 0.753 (hazard ratio [HR], 1.132 [95% CI, 0.522–2.457]) (Table 3) and 0.976 (HR, 1.019 [95% CI, 0.300–3.464]) (Table 4), respectively.

**Table 3:** Multivariant analyses of progression free survival

	95%CI			
	HR	Lower	Upper	p
Gender	1.996	0.823	4.842	0.127
ECOG	1.610	0.344	7.534	0.545
Smoking history	0.981	0.397	2.425	0.967
MWA	1.132	0.522	2.457	0.753
Response	0.619	0.295	1.301	0.206

**Table 4:** Multivariant analyses of progression free survival

	95%CI			
	HR	Lower	Upper	p
Gender	2.082	0.622	6.966	0.234
Age	1.564	0.538	4.552	0.412
EGFR mutations	0.478	0.155	1.473	0.199
MWA	1.019	0.300	3.464	0.976
Response	0.280	0.087	0.900	0.033
Post-EGFR-TKIs	0.404	0.121	1.343	0.139

**Figure 3:**
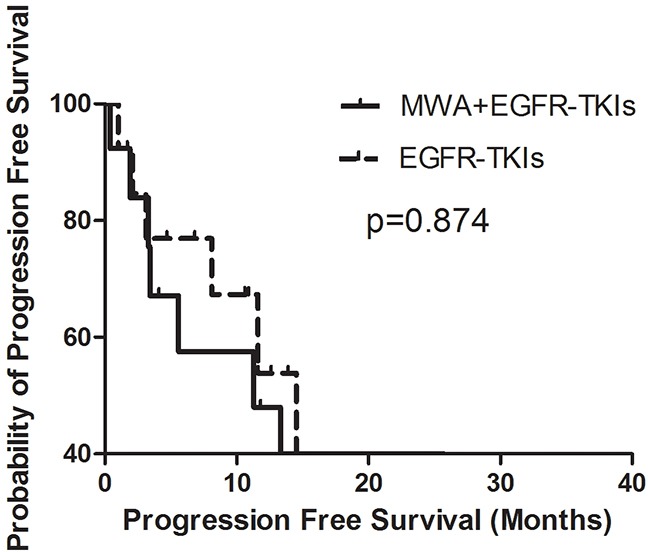
Kaplan-Meier estimates of PFS in 26 patients with metastatic sites more than 3 The median PFS of patients treated with MWA plus EGFR-TKIs was 11.3 months (95%CI, 3.1-22.9 months), and those received EGFR-TKIs was 15.1 months (95%CI, 8.1-22.2 months).

**Figure 4:**
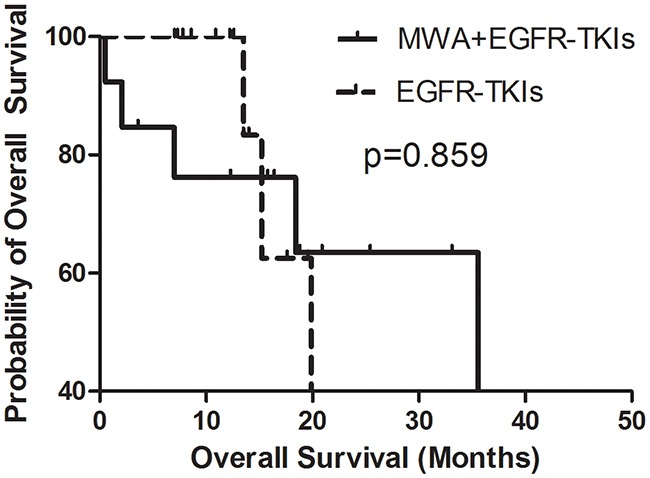
Kaplan-Meier estimates of OS in 26 patients with metastatic sites more than 3 The median OS of patients treated with MWA plus EGFR-TKIs was 28.4 months (95%CI, 18.1-38.6 months), and those received EGFR-TKIs was 18.2 months (95%CI, 15.1-21.2 months).

**Figure 5:**
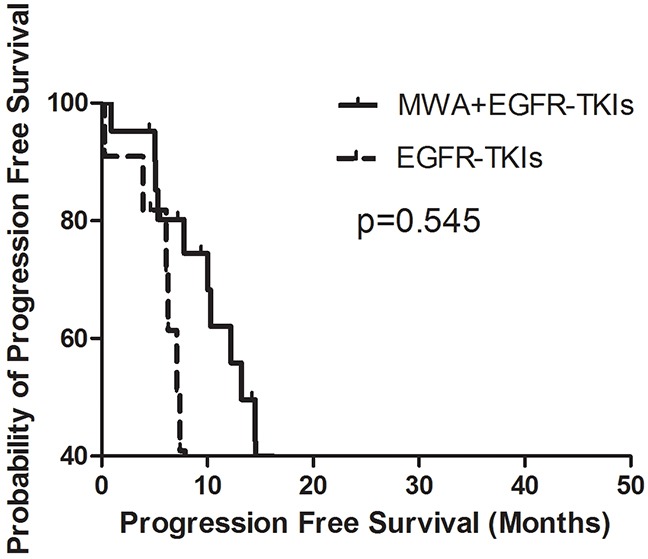
Kaplan-Meier estimates of PFS in 32 patients with metastatic sites of 3 or fewer The median PFS of patients treated with MWA plus EGFR-TKIs was 13.2 months (95%CI, 9.1-17.4 months), and those received EGFR-TKIs was 7.4 months (95%CI, 5.7-9.1 months).

**Figure 6:**
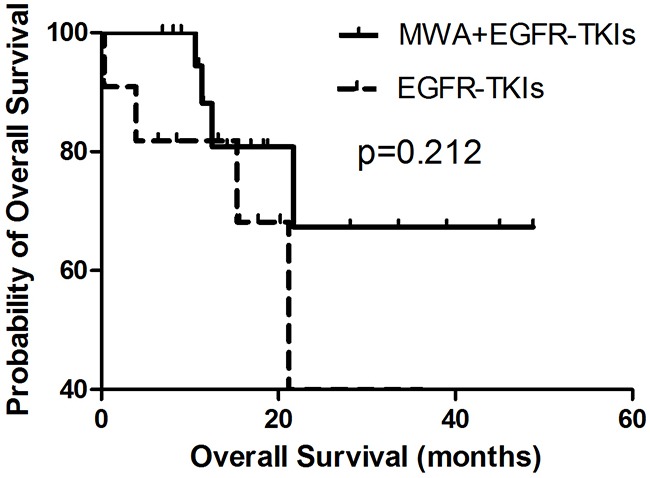
Kaplan-Meier estimates of OS in 32 patients with metastatic sites of 3 or fewer The median OS of patients treated with MWA plus EGFR-TKIs was 38.0 months (95%CI, 28.8-47.2 months), and those received EGFR-TKIs was 23.3 months (95%CI, 12.2-34.3 months).

In order to explore the correlation between the number of tumor sites and the survival benefit of MWA, we divided patients into two groups, those with three or fewer tumor metastases and those with more than three tumor sites other than the primary tumor in advanced. No differences were observed in either group in PFS or OS (Figures 3, 4, 5, 6). For patients with more than three tumor metastases, the median PFS were 11.3 months (95% CI, 3.1–22.9 months) and 15.1 months (95% CI, 8.1–22.2 months) (p = 0.874) (Figure 3) for the MWA plus EGFR-TKIs group and the EGFR-TKIs group, respectively, and the corresponding OS were 28.4 months (95% CI, 18.1–38.6 months) and 18.2 months (95% CI, 15.1–21.2 months) (p = 0.859), respectively (Figure 4). For patients with three or fewer tumor metastases, the median PFS were 13.2 months (95% CI, 9.1–17.4 months) and 7.4 months (95% CI, 5.7–9.1 months) (p = 0.545) (Figure 5) for the MWA plus EGFR-TKIs group and the EGFR-TKIs group, respectively, and the corresponding OS were 38.0 months (95% CI, 28.8–47.2 months) and 23.3 months (95% CI, 12.2–34.3 months), respectively (p = 0.212) (Figure 6).

## DISCUSSION

EGFR-TKIs remain the standard of care for advanced NSCLC with EGFR-sensitive mutations and show a dramatic improvement in PFS. [1–5] However, all patients will develop resistance ultimately, even those who achieve complete or incomplete remission. [6–18]

Multiple studies explored the following treatment strategy after the failure of EGFR-TKIs. Based on the progression models, the therapies varied. [19, 20] For those with brain progression, SBRT or SRT was the first choice, and the EGFR-TKIs were continued. [20–22] For patients with local disease progression, local treatments including radiation or thermal ablation in combination with previous EGFR-TKIs were recommended [20, 23, 24, 33–35]. However, chemotherapy and transfer to a third EGFR-TKI were treatment options for those with widespread metastasis.

We firstly explored whether the combination of MWA at primary tumor sites and EGFR-TKIs could improve PFS when compared with EGFR-TKIs alone for advanced NSCLC with EGFR-sensitive mutations. No significant difference was observed in either PFS or OS in univariate and multivariate analyses, indicating that MWA and EGFR-TKIs did not have a synergistic effect, which was different from radiation plus EGFR-TKIs. Welsh et al. [21] showed that patients with EGFR-sensitive mutations and brain metastases had superior survival and ORRs when treated with EGFR-TKIs and SBRT. William et al. [22] verified that the median OS and intracranial PFS were longer in the group treated with RT upfront compared with the group treated with EGFR-TKIs upfront. What is more, Zeng et al. [36] showed that concomitant administration of gefitinib and WBRT was found to result in higher treatment response and disease control rates in patients with EGFR-sensitive mutations and NSCLC brain metastases compared with gefitinib alone. Hong et al. [33] found that in NSCLC patients with EGFR-sensitive mutations and skeletal metastasis progression, EGFR-TKIs continued in combination with radiation after treatment with EGFR-TKIs and disease progression resulted in a PFS of 5.6 to 8.0 months. Isolated bone failure without systemic disease progression is associated with better survival when treated with continuation of EGFR-TKIs plus local radiation. [24] Studies verified that EGFR-TKIs could modulate the radiation response. [37, 38] When combined with radiation, EGFR-TKIs promote a further reduction in the S-phase fraction, resulting in an accumulation of cells in G1 and G2. [37] What is more, TKIs enhance the induction of apoptosis, inhibit EGFR autophosphorylation and Rad51 expression, and improve radiosensitivity. [37] EGFR inhibition led to pronounced cellular senescence of irradiated cells. Moreover, cellular senescence is a prominent mechanism in radiosensitization. The senescence and radiosensitization were linked to an increase in residual radiation-induced DNA double-strand breaks irrespective of p53/p16 status. [39]

To explore the correlation between the number of tumor sites and the survival benefit of MWA, we divided patients into two groups, those with three or fewer tumor metastases and those with more than three tumor sites other than the primary tumor in advanced. No differences were observed in either group in PFS or OS. Gomez et al. [28] showed that advanced NSCLC patients without driver mutations after systematic treatments of platinum-based doublet chemotherapy and patients with driver mutations after EGFR-TKIs or ALK inhibitors had longer PFS when treated with consolidated treatments followed by maintenance treatments compared with maintenance treatments alone. An 8-month PFS was observed, which was significantly different. However, the study was restricted to those with oligometastases, which was defined as no more than three tumor sites. Chiang et al. [34] also showed that radical palliative thoracic RT was safe and might be beneficial for primary lung lesions in patients with metastatic NSCLC and controlled extrathoracic diseases. The median OS, PFS after RT, and OS after RT were 50 months, 15 months, and 18 months, respectively. Qin et al. [35] reported that when patients who responded to EGFR-TKIs received EGFR-TKIs and local radiofrequency hyperthermia, the PFS and OS were 22 months and 26 months, respectively. The difference in PFS indicated that local treatments, which did not focus on all tumor sites, did not have a survival advantage for patients with EGFR-sensitive mutations treated with MWA plus EGFR-TKIs.

In conclusion, for advanced NSCLC patients with EGFR-sensitive mutations, MWA at the primary tumor sites plus EGFR-TKIs failed to show a survival advantage when compared with EGFR-TKIs alone.

## MATERIALS AND METHODS

### Patient characteristics

Patients with pathologically verified advanced NSCLC and EGFR-sensitive mutations, i.e., an exon 19 deletion or exon 21 L858R point mutation, were recruited. Other inclusion criteria included an age no less than 18 years; Eastern Cooperative Oncology Group Performance Status (ECOG PS) of 0 to 1; no local therapy at primary tumor sites, such as radiation, radiofrequency ablation (RFA), or I125 radioactive particle implantation; and adequate bone marrow, hepatic, and renal function. The exclusion criteria were as follows: serious issues with pulmonary and cardiovascular function such that MWA could not be risked; previous treatment with EGFR-TKIs or local therapies for primary tumors; baseline interstitial lung disease; cerebral hemorrhage, cerebral infarction, or coronary heart disease, especially unstable angina or myocardial infarction during the previous 6 months; and anti-platelet or anti-necrosis treatments during the past 1 week.

The study was approved by the ethics committee of Shandong Provincial Hospital affiliated with Shandong University prior to study enrollment. Written informed consent was obtained from all enrolled patients.

### MWA procedure

All patients allocated to the MWA plus EGFR-TKIs group received MWA at primary tumor sites under computed tomography guidance. The procedure has been detailed in our previous reports. [25, 26, 29]

### EGFR mutation testing

DNA was extracted from 4-μm formalin-fixedand parrffin-embedded,(FFPE) tumor slides by using the QIAamp DNA FFPE Tissue Kit (Qiagen, Germany) according to the manufacturer's instructions. The EGFR mutation testing procedure was described in detail in our previous study. [30] A classic S-curve and a Ct value >30 were considered to be a positive result, indicating the presence of a mutation.

### EGFR-TKIs

Patients received 250 mg oral gefitinib or 150 mg erlotinib once daily until disease progression or intolerable toxicity. The interval between MWA and EGFR-TKIs therapy was 1 week.

### The follow-up and response evaluation

MWA follow-up was performed every month for 3 months post-ablation and at 3-month intervals thereafter. The response to EGFR-TKIs was evaluated 1 month later and then at 2-month intervals.

The response to MWA was assessed according to a Chinese expert consensus [31]. The response to EGFR-TKIs was conducted according to the Response Evaluation Criteria in Solid Tumors (RECIST) version 1.1 [32].

### Statistical analyses

SPSS version 17.0 (USA) was applied for statistical analyses. The primary endpoint was PFS; the secondary endpoint was overall survival (OS). PFS was calculated from the date of MWA to disease progression for both primary tumor sites and other tumor sites or death for those treated with MWA plus EGFR-TKIs. For patients who received EGFR-TKIs only, PFS was calculated from the start of treatment with EGFR-TKIs to disease progression or death. OS was calculated from the diagnosis of NSCLC to death from any cause. Both PFS and OS were analyzed using Kaplan-Meier univariate analyses and Cox regression multivariate analyses. Factors with p-values less than 0.5 in the univariate analyses besides MWA were examined in the Cox regression multivariate analyses. Chi-square test was used to test the correlation between the response to EGFR-TKIs and the treatment regimens. All tests were two-sided, and p-values less than 0.05 were considered to represent a significant difference.
